# Changes in Couples’ Relationships and Their Differences in Type during the COVID-19 Pandemic in China

**DOI:** 10.3390/ijerph191912516

**Published:** 2022-09-30

**Authors:** Qi Jiang

**Affiliations:** Police Academy, Shandong University of Political Science and Law, Jinan 250014, China; jiangq@sdupsl.edu.cn

**Keywords:** COVID-19, couples’ relationships, positive communication, criticism/defense, demand/withdrawal

## Abstract

Purpose: This study explores changes in couples’ relationships during the COVID-19 pandemic, and analyzes the differences in the changes across three types: positive communication, criticism/defense, and demand/withdrawal. Method: A total of 600 (567 valid) Chinese respondents participated in this study, and a questionnaire was utilized to determine changes in their overall relationship, verbal and nonverbal communication, emotion, and activities with their spouses. Results: The average score of items related to positive communication is higher, compared with that of negative communication. Compared with the other two types of relationships, respondents with positive communication scored highest on all items related to positive communication and lowest on all items related to negative communication. Significant differences were noted between the positive communication types and the others. Conclusions: Results show that the relationships of couples included in this study have improved during the current pandemic. Therefore, improved consistency in the type of intimacy can lead to improved quality of couples’ relationships during the COVID-19 pandemic.

## 1. Introduction

External stress can negatively affect couples’ relationships. When faced with stressful situations, such as unemployment, poverty, or work distress, people are likely to blame their spouses, neglect their spouses’ needs, and disagree with their spouses [1,2,3,4].

As an external stress situation, disasters may negatively impact couples’ relationships. Cohan and Cole [5] found that counties in South Carolina that were deemed disaster areas during Hurricane Hugo noted increased marriage, divorce, and birth rates in the second year compared with other counties in the state. Chan and Zhang [6] noted that all types of domestic violence, including psychological attacks and physical violence, between couples had increased after the 12 May 2008 Sichuan Earthquake. Buttell and Carney [7] and Enarson and Scanlon [8] also found that interpersonal violence and abuse becomes more severe postdisaster.

Disasters can also positively affect couples’ relationships. Caruana [9] noted increased family-cohesion levels during the postdisaster recovery period. In the aftermath of both the September 11 and Oklahoma terrorist attacks in the United States, divorce rates in surrounding areas decreased [10,11].

Different effects may reflect the different backgrounds of these two types of disasters [5], which led to the differentiation of couples’ relationships in this study. Natural disasters and their consequences are deterministic. Rebuilding homes can cause strenuous and long-term stress on marriage and family, thereby leading to increased divorce rates. However, terrorist attacks are related to major loss of life and can cause uncertainty and fears about future attacks. When faced with terrorist threats, people typically seek physical contact, safety, and comfort with their spouses.

The COVID-19 pandemic presents a particularly intense external stress, and research has yet to explore its impact on couples’ relationships. However, this pandemic has presented common characteristics of natural disasters (e.g., the impact will last for months or even years after the pandemic) and terrorist attacks (e.g., many people have lost their lives, and uncertainty and fear are widespread among the world population). The first characteristic has led to an increase in the divorce rate, whereas the second characteristic has led to a decrease in the divorce rate. To confirm whether the divorce rate is increasing or decreasing, the type of communication before the crisis occurred must be examined.

Research has presented many perspectives on the type of communication between couples. Focusing on “conflict,” Pruitt [12] noted four types of communication: problem-solving (concern for both), contending (concern for self), yielding (concern for other), or inaction (concern for neither). Based on their attachment style, couples in a relationship may resolve (constructive engagement), intensify (destructive engagement), or avoid the conflict (conflict avoidance). Dickson [13] identified three types of couples in long-lasting marriages: connected couples (closeness, intimacy, and dependence); functional separate couples (support each other and remain independent); and dysfunctional separate couples (distance and dissatisfaction with the marriage). Cohen et al. [14] noted three types of enduring marriages: vitalized, satisfactory, and conflictual. Marks [15] proposed four high-quality and three low-quality marriage types that differed based on the manner in which each spouse balanced the energy they placed in their personal interiority. Rauer and Volling [16] revealed the existence of three types of couples based on observed behaviors in a problem-solving task: (1) mutually engaged couples (characterized by both spouses’ higher negative and positive problem-solving); (2) mutually supportive couples (characterized by both spouses’ higher positivity and support); and (3) wife compensation couples (characterized by high wife positivity).

To summarize, these can be categorized into three types: positive, compromise, and negative. Therefore, the classification types used in this study are positive communication, demand/withdrawal, and criticism/defense [17]. Positive communication indicates that couples will actively express their feelings and are willing to discuss existing problems, which is conducive to the development of the relationship. The demand/withdrawal pattern has been consistently noted to be detrimental for marital satisfaction [18]. In this pattern, one person attempts to approach their partner about a problem, perhaps even through nagging and complaining, and their partner denies the existence of the problem through avoidance, denial, refusal to discuss it, and so on [19,20]. Criticism/defense is a common negative communication wherein couples shirk each other’s responsibilities, clarify themselves, and blame each other, thereby leading to a significantly negative effect on the marriage.

Researches showed that during the pandemic, the relationship satisfaction of the couples engaged in positive coping was enhanced, and the attribution of maladaptation was decreased. In relationship-conflict couples, the result was just the opposite [21]. Positive and effective communication can mitigate the impact of COVID-19 on relationships and is a good opportunity for relationships to grow [22]. It is necessary to explore the impact of the pandemic on couples’ relationship groups in different cultures and regions [23]. A German-based study [24] showed that in the short term, couples’ relationships were not affected by the lockdown measures and required long-term observation and tracking. Negative interactions between Israeli couples aggravated the impact of stress on negative emotions [25]. The quality of Kenyan couples’ sexual relationships declined during the pandemic [26]. The COVID-19 pandemic should be considered an etiology of sexual dissatisfaction and possibly sexual dysfunctions, and similar findings have appeared in Italy [27]. Research on Chinese people is limited [28,29].

This study aimed to investigate changes in Chinese couples’ relationships and differences in the types of change during the COVID-19 pandemic. In early 2020, especially in January, the number of people infected with COVID-19 in China rose sharply, so from about the end of January, society began to stop work and production. People had to stay at home and quarantined, reducing a large number of outbound activities: closure of schools, curfews, bans on weddings and funerals, etc. It was not until early May that the pandemic was well controlled and society began to return to work, when the data collection of this study was carried out.

## 2. Methods

### 2.1. Respondents

Using convenience and snowball sampling, a questionnaire was distributed online to measure Chinese respondents who lived together in couples’ relationships (just being in a relationship, even without marriage) in Shandong province. A total of 600 questionnaire responses were collected, and the following samples were excluded: responses wherein respondents had taken too long (>900 s) or too less time (<120 s) to respond, and poor-quality responses were removed. The final number of respondents was 567, with an effective recovery rate of 94.5%. In terms of gender, 243 (42.86%) respondents were male and 324 (57.14%) respondents were female. In terms of education, 53 respondents (9.35%) had achieved a junior high school diploma, 32 (5.64%), a high school diploma, 51 (8.99%), an associate degree, 262 (46.21%), a bachelor’s degree, and 169 (29.81%), a graduate degree. A total of 440 (77.6%) respondents had children and 127 (22.4%) had no children. In terms of jobs, 105 (18.25%) respondents were medical workers, 266 (46.91%), stable professionals, and 196 (34.57%) unstable professionals. A total of 389 (68.61%) respondents were urban residents and 178 (31.39%) respondents were rural residents. Among the respondents, 256 (45.15%) reported positive communication, 106 (18.69%) criticism/defense, and 205 (36.16%) demand/withdrawal patterns. In terms of age, 54 (9.52%) respondents were aged 18–25 years, 300 (52.91%) 26–35 years, 178 (31.39%) 36–50 years, 32 (5.64%) 51–65 years, and 3 (0.53%) above 65 years. Finally, 552 (97.35%) respondents were of Han ethnicity and 15 (2.65%) respondents were minorities.

### 2.2. Procedure

Data were collected online using the WeChat software. The tool used for data analysis was SPSS 22.0. Respondents were quarantined at home from February to April 2020. The study was conducted during the first week of May 2020. At that time, China’s pandemic had been brought under control, and social life had gradually returned to stability.

### 2.3. Tools

The self-edited “Questionnaire on Changes in couples’ relationship during the COVID-19 pandemic” was distributed to respondents. The questionnaire has not been published anywhere, nor is it adapted from any previous questionnaire. The questionnaire was divided into six parts, with a total of 45 questions (α = 0.824).

#### 2.3.1. General Changes

This part included a description of couples’ advantages, weaknesses, feelings, and attractiveness, and a total of four questions.

#### 2.3.2. Changes in Verbal Communication with One’s Spouse

This part inquired about volume of verbal communication (one question), positive verbal communication (four questions), negative verbal communication (four questions), and spiritual communication (four questions), for a total of thirteen questions.

#### 2.3.3. Changes in Nonverbal Communication with One’s Spouse

This part inquired about positive physical contact (four questions), negative physical contact (two questions), facial expression (two questions), for a total of eight questions.

#### 2.3.4. Emotional Changes Related to One’s Spouse

This part inquired about positive emotions (two questions) and negative emotions (six questions), with a total of eight questions.

#### 2.3.5. Activities with One’s Spouse

This part inquired about housework, cooking, reading, and exercise, with a total of four questions.

In addition to the sociodemographic survey, all other items were scored out of three, wherein 1 point indicated deterioration/decrease, 2 points indicated no change, and 3 indicated improvement/increase. For example, Q9: During the COVID-19 pandemic, I think his/her advantages have been ()?

## 3. Results

### 3.1. Changes in Couples’ Relationships during the COVID-19 Pandemic (See Table 1)

In Table 1, the basic situation of respondents in sociodemographic variables such as gender, education, have children or not, occupation, region, consistent type of communication, age, and ethnicity are introduced.

**Table 1:** Sociodemographic Variables.

	Number	Proportion
Q1 Gender		
Male	243	42.86%
Female	324	57.14%
Q2 Education		
Junior high school diploma	53	9.35%
High school diploma	32	5.64%
Associate degree	51	8.99%
Bachelor’s degree	262	46.21%
Graduate degree	169	29.81%
Q3 Have children or not		
Yes	440	77.6%
No	127	22.4%
Q4 Occupation		
Medical workers	105	18.52%
Stable professional	266	46.91%
Unstable professional	196	34.57%
Q5 Region		
Urban	389	68.61%
Rural	178	31.39%
Q6 Consistent type of communication		
Positive communication	256	45.15%
Criticism/defense	106	18.69%
Demand/withdrawal	205	36.16%
Q7 Age		
18–25	54	9.52%
26–35	300	52.91%
36–50	178	31.39%
51–65	32	5.64%
Over 65	3	0.53%
Q8 Ethnicity		
Han	552	97.35%
Minorities	15	2.65%

#### 3.1.1. General Changes (See Table 2)

The results in Table 2 show that the average scores for positive evaluations of spouses were higher (Q9, Q11, Q12), whereas that for negative evaluations was lower (Q10). This result showed that intimate relationships may become more active during home quarantine.

**Table 2:** General Changes in Couples’ Relationships.

	Deteriorated/Decreased	Unchanged	Improved/Increased	$\bar{X}$ ± *s*
Items				
Q9 His/her advantages	40 (7.05%)	207 (36.51%)	320 (56.44%)	2.49 ± 0.63
Q10 His/her weaknesses	144 (25.4%)	318 (56.08%)	105 (18.52%)	1.93 ± 0.66
Q11 Feelings between us	32 (5.64%)	258 (45.5%)	277 (48.85%)	2.43 ± 0.60
Q12 His/her attractiveness	38 (6.7%)	313 (55.2%)	216 (38.1%)	2.31 ± 0.59

#### 3.1.2. Changes in Verbal Communication (See Table 3)

In Table 3, the average scores for volume of verbal communication (Q13), positive verbal communication (Q14–17), and spiritual communication (Q22–25) were relatively high, whereas that for negative verbal communication (Q18–20) was low. This result showed that verbal communication between couples had become more active and verbal conflicts had decreased.

**Table 3:** Changes in Verbal Communication within Couples.

	Deteriorated/Decreased	Unchanged	Improved/Increased	$\bar{X}$ ± *s*
Items				
Q13 Volume of talk	39 (6.88%)	212 (37.39%)	316 (55.73%)	2.49 ± 0.62
Q14 Encourage each other	25 (4.41%)	256 (45.15%)	286 (50.44%)	2.46 ± 0.58
Q15 Care for each other	26 (4.59%)	204 (35.98%)	337 (59.44%)	2.55 ± 0.58
Q16 Affirm each other	27 (4.76%)	253 (44.62%)	287 (50.62%)	2.46 ± 0.59
Q17 Praise each other	27 (4.76%)	277 (48.85%)	263 (46.38%)	2.42 ± 0.58
Q18 Belittle each other	152 (26.81%)	346 (61.02%)	69 (12.17%)	1.85 ± 0.61
Q19 Blame each other	159 (28.04%)	331 (58.38%)	77 (13.58%)	1.86 ± 0.63
Q20 Ironize each other	166 (29.28%)	338 (59.61%)	63 (11.11%)	1.82 ± 0.61
Q21 Insult each other	169 (29.81%)	371 (65.43%)	27 (4.76%)	1.75 ± 0.53
Q22 Frequency of spiritual communication	30 (5.29%)	244 (43.03%)	293 (51.68%)	2.46 ± 0.60
Q23 Depth of spiritual communication	30 (5.29%)	263 (46.38%)	274 (48.32%)	2.43 ± 0.59
Q24 Compatibility of spiritual communication	26 (4.59%)	271 (47.8%)	270 (47.62%)	2.43 ± 0.58
Q25 Willingness of spiritual communication	28 (4.94%)	263 (46.38%)	276 (48.68%)	2.44 ± 0.59

#### 3.1.3. Changes in Nonverbal Communication (See Table 4)

In Table 4, the average scores for positive physical contact (Q26–29) and facial expression (Q32–33) between couples were relatively high, whereas that for negative physical contact (Q30–31) was low. This result showed that nonverbal communication between couples improved, whereas physical conflicts decreased.

**Table 4:** Changes in Nonverbal Communication with Spouse.

	Deteriorated/Decreased	Unchanged	Improved/Increased	$\bar{X}$ ± *s*
Items (Frequency)				
Q26 Hold hands	72 (12.7%)	318 (56.08%)	177 (31.22%)	2.19 ± 0.64
Q27 Hugs	61 (10.76%)	302 (53.26%)	204 (35.98%)	2.25 ± 0.64
Q28 Kiss	74 (13.05%)	317 (55.91%)	176 (31.04%)	2.18 ± 0.64
Q29 Sex	77 (13.58%)	328 (57.85%)	162 (28.57%)	2.15 ± 0.63
Q30 Physical conflicts (such as beating, pushing, etc.)	134 (23.63%)	381 (67.2%)	52 (9.17%)	1.86 ± 0.56
Q31 Other acts of violence	124 (21.87%)	405 (71.43%)	38 (6.7%)	1.85 ± 0.51
Q32 Eye contact	44 (7.76%)	284 (50.09%)	239 (42.15%)	2.34 ± 0.62
Q33 Exude tenderness and love through eyes	33 (5.82%)	200 (35.27%)	334 (58.91%)	2.53 ± 0.61

#### 3.1.4. Changes in Emotion (See Table 5)

In Table 5, the average scores of positive emotions (Q34–35) between couples were relatively high, whereas that of negative emotions (Q36–41) was low. This result showed that positive emotions among couples increased, whereas negative emotions decreased.

**Table 5:** Changes in Emotion.

	Deteriorated/Decreased	Unchanged	Improved/Increased	$\bar{X}$ ± *s*
Items				
Q34 Happy	44 (7.76%)	210 (37.04%)	313 (55.2%)	2.47 ± 0.64
Q35 Security	42 (7.41%)	205 (36.16%)	320 (56.44%)	2.49 ± 0.63
Q36 Annoying	253 (44.62%)	260 (45.86%)	54 (9.52%)	1.65 ± 0.65
Q37 Angry	245 (43.21%)	260 (45.86%)	62 (10.93%)	1.68 ± 0.66
Q38 Sad	245 (43.21%)	274 (48.32%)	48 (8.47%)	1.65 ± 0.63
Q39 Hate	277 (48.85%)	265 (46.74%)	25 (4.41%)	1.56 ± 0.58
Q40 Disappointment	254 (44.8%)	260 (45.86%)	53 (9.35%)	1.65 ± 0.65
Q41 Fear	283 (49.91%)	260 (45.86%)	24 (4.23%)	1.54 ± 0.58

#### 3.1.5. Changes in Activities (See Table 6)

In Table 6, the average scores of activities (Q42–45) that couples participated in together were relatively high. This result indicated that the possibility of partners doing things together was increasing.

**Table 6:** Changes in Activities with Spouse.

	Deteriorated/Decreased	Unchanged	Improved/Increased	$\bar{X}$ ± *s*
Items				
Q42 Housework	24 (4.23%)	209 (36.86%)	334 (58.91%)	2.55 ± 0.58
Q43 Cooking	30 (5.29%)	209 (36.86%)	328 (57.85%)	2.53 ± 0.60
Q44 Reading	31 (5.47%)	330 (58.2%)	206 (36.33%)	2.31 ± 0.57
Q45 Exercise	32 (5.64%)	259 (45.68%)	276 (48.68%)	2.43 ± 0.60

### 3.2. Differences between Three Types of Couples’ Relationship

#### 3.2.1. Changes in General Changes (See Table 7)

By conducting one-way ANOVA (Table 7), in the positive evaluation of overall changes in couples’ relationships, the average scores in the positive-communication group were significantly higher than the other two types. In the negative evaluation, the average score was also significantly lower. Therefore, more positive types of communication between couples led to increasingly positive evaluations of changes in their relationships.

**Table 7:** Differences in General Changes.

	Consistent Type of Communication’ (*C*)			*F*	*P*	Multiple Comparisons	
Items	Criticism /Defense	Demand /Withdrawal	Positive Communication			Positive Communication	
						Criticism /Defense	Demand /Withdrawal
Q9 his/her advantages	2.29	2.39	2.66	19.19	0.000	0.000	0.000
Q10 his/her weaknesses	2.19	1.96	1.80	13.89	0.000	0.000	0.02
Q11 feelings with him/her	2.22	2.40	2.55	12.76	0.000	0.000	0.01
Q12 his/her attractiveness	2.12	2.29	2.41	9.70	0.000	0.000	0.02

#### 3.2.2. Changes in Verbal Communication (See Table 8)

Table 8 shows that on all the items of verbal communication, the positive-communication group showed very significant differences from the other two types. Better consistency in the type of communication between couples led to higher scores for positive communication and spiritual communication during home quarantine, and lower scores for negative communication. This finding indicated that a consistently good relationship between couples may be the basis for good communication in the face of external pressure in the future.

**Table 8:** Differences in Verbal Communication with Spouse.

	Consistent Type of Communication’ (*C*)			*F*	*P*	Multiple Comparisons	
Items	Criticism /Defense	Demand /Withdrawal	Positive Communication			Positive Communication	
						Criticism /Defense	Demand /Withdrawal
Q13 Volume of talk	2.34	2.43	2.59	7.64	0.001	0.003	0.02
Q14 Encourage each other	2.28	2.36	2.61	17.82	0.000	0.000	0.000
Q15 Care for each other	2.36	2.49	2.68	13.40	0.000	0.000	0.001
Q16 Affirm each other	2.28	2.38	2.60	14.67	0.000	0.000	0.001
Q17 Praise each other	2.25	2.34	2.54	12.31	0.000	0.000	0.000
Q18 Belittle each other	2.00	1.91	1.75	8.25	0.000	0.003	0.007
Q19 Blame each other	1.99	1.92	1.75	7.63	0.001	0.007	0.006
Q20 Ironize each other	1.96	1.89	1.70	9.66	0.000	0.002	0.001
Q21 Insult each other	1.86	1.82	1.65	8.84	0.000	0.004	0.001
Q22 Frequency of spiritual communication	2.34	2.38	2.59	10.25	0.000	0.000	0.000
Q23 Depth of spiritual communication	2.31	2.31	2.58	15.23	0.000	0.000	0.000
Q24 Compatibility of spiritual communication	2.28	2.30	2.60	20.73	0.000	0.000	0.000
Q25 Willingness of spiritual communication	2.30	2.31	2.59	17.49	0.000	0.000	0.000

#### 3.2.3. Changes in Nonverbal Communication (See Table 9)

In Table 9, in terms of positive physical contact, with the deepening of the degree of communication, differences between the positive-communication group and the other two types became less notable. One potential explanation is that holding hands and hugging can be easily controlled by people; however, sexual needs are closely related to physical impulses, and psychological factors can be ignored and satisfaction can be achieved first. In terms of negative physical communication, the positive-communication group showed no difference only in fierce physical conflict from the criticism/defense type. A potential reason for this finding may be that the significant harm of fierce physical conflict only occurs when the intimate relationship has severely deteriorated. In terms of facial expression communication, the positive-communication group showed very significant differences from the other two types.

**Table 9:** Differences in Nonverbal Communication with Spouse.

	Consistent Type of Communication’ (*C*)			*F*	*P*	Multiple Comparisons	
Items	Criticism /Defense	Demand /Withdrawal	Positive Communication			Positive Communication	
						Criticism /Defense	Demand /Withdrawal
Q26 Hold hands	2.00	2.17	2.28	7.42	0.001	0.000	0.18
Q27 Hugs	2.14	2.23	2.32	3.07	0.047	0.04	0.38
Q28 Kiss	2.07	2.19	2.22	2.27	0.10		
Q29 Sex	2.11	2.15	2.16	0.24	0.79		
Q30 Physical conflicts (such as beating, pushing, etc.)	1.89	1.92	1.79	3.08	0.047	0.41	0.045
Q31 Other acts of violence	1.92	1.89	1.79	3.64	0.03	0.03	0.03
Q32 Eye contact	2.15	2.29	2.46	11.17	0.000	0.000	0.003
Q33 Exude tenderness and love through eyes	2.36	2.47	2.65	10.40	0.000	0.000	0.004

#### 3.2.4. Changes in Emotion (See Table 10)

Studies have shown that emotions/affect are the most valuable observation indicators when discussing conflicts in couples’ relationships [30]. The results in Table 10 show that on all items of emotional changes, the positive-communication group showed very significant differences from the other two types. Better consistency in the type of communication between couples led to higher scores for positive emotions and lower scores for negative emotions during the pandemic. This finding suggested that a consistently good relationship between couples may have a protective effect on positive emotions and defensive effect on negative emotions.

**Table 10:** Differences in Emotional Changes.

	Consistent Type of Communication’ (*C*)			*F*	*P*	Multiple Comparisons	
Items	Criticism /Defense	Demand /Withdrawal	Positive Communication			Positive Communication	
						Criticism /Defense	Demand /Withdrawal
Q34 Happy	2.25	2.38	2.64	18.33	0.000	0.000	0.000
Q35 Security	2.27	2.41	2.64	15.75	0.000	0.000	0.000
Q36 Annoying	1.89	1.70	1.51	14.11	0.000	0.000	0.002
Q37 Angry	1.90	1.89	1.54	12.71	0.000	0.000	0.001
Q38 Sad	1.88	1.74	1.49	18.59	0.000	0.000	0.000
Q39 Hate	1.76	1.74	1.42	16.16	0.000	0.000	0.000
Q40 Disappointment	1.92	1.74	1.46	24.28	0.000	0.000	0.000
Q41 Fear	1.72	1.59	1.44	9.94	0.000	0.000	0.01

#### 3.2.5. Changes in Activities (See Table 11)

Based on the results in Table 11, it can be noted that there was no significant difference only for cooking (Q41) between the positive-communication and demand/withdrawal groups. For other shared activities, the positive-communication group showed significant differences from the other two types. Better consistency in the type of communication between couples leads to increased ease in the participation of activities together. The results also showed that scores of all types of people on these five items were high, which may be related to space constraints, and it is difficult to avoid having overlapping activities between couples.

**Table 11:** Differences in Activities with Spouse.

	Consistent Type of Communication’ (*C*)			*F*	*P*	Multiple Comparisons	
Items	Criticism /Defense	Demand /Withdrawal	Positive Communication			Positive Communication	
						Criticism /Defense	Demand /Withdrawal
Q42 Housework	2.44	2.49	2.63	50.53	0.004	0.02	0.03
Q43 Cooking	2.38	2.51	2.60	50.27	0.005	0.001	0.12
Q44 Reading	2.17	2.25	2.41	80.94	0.000	0.001	0.005
Q45 Exercise	2.32	2.39	2.51	40.78	0.01	0.006	0.02

## 4. Discussion

### 4.1. Relationships Are Developing in a Better Direction

Questions related to positive communication in the questionnaire had higher average scores than those related to negative communication. All items related to the theme of “active coexistence” had higher scores. This result shows that during the early stage of the COVID-19 pandemic, positive communication in couples’ relationships was more popular, and relationships between couples were developing in a better direction. This finding was inconsistent with some news reports that home quarantine may lead to an increase in divorce rate during the early stage of the COVID-19 pandemic. We searched for keywords, such as “pandemic” and “divorce rate,” on China National Knowledge Infrastructure, and found no direct research evidence to support these news reports. Researchers have also observed that many survivors of disasters and other traumatic events experience improved interpersonal relationships [31].

People have a fundamental need for a sense of belonging. When they perceive a close relationship with important others, they are most likely to thrive when faced with pressure [32]. During home quarantine, people have fewer opportunities to meet friends and relatives, and this has provided couples with more opportunities for communication and exchange in terms of both space and time. Moreover, coupled with the atmosphere of fear caused by the pandemic, it is easier to seek comfort and support from couples.

### 4.2. Differences in Relationship Types

This study found that better consistency in the type of intimacy led to improved quality of couples’ relationships during the early stage of the COVID-19 pandemic. For most of the items, the changes in relationship reported by the positive-communication couples were better than those with criticism/defense and demand/withdrawal patterns: (1) changes in verbal communication (positive, negative, and spiritual communication); (2) changes in nonverbal communication (positive and negative physical contact and facial expression); (3) emotional changes (positive and negative); and (4) activities with couples. It must be noted that for Q29 and Q30, no significant differences were found. The reason may be that kissing and sexual behavior, as behaviors closer to animal nature, have more to do with biological instincts.

In negative couples’ relationships (criticism/defense and demand/withdrawal pattern), individuals under stress have decreased ability to offer support to their spouses [33,34], and thus stressors can interfere with a couples’ ability to work jointly toward alleviating stress. They are prone to be trapped in negative communication cycles that are difficult to break [35,36]. In contrast, people in positive marital and couples’ relationships are able to frame their relationship distress as external and temporary, engage in joint problem-solving, express support, and accept faults in their relationship [22,37,38,39]. Happy couples maintain a 5:1 ratio of positive to negative behaviors even during conflict discussions, significantly higher than that of couples experiencing unhappy marriages [40,41]. The results of this study verified these perspectives.

Therefore, if both couples work hard to maintain intimate relationships, it is possible to overcome the negative effects of external pressure. For example, accepting the spouse’s occasional criticism, forgiving their injury, reducing the expression of blame, hostility, and contempt, and participating in light and lively activities can enhance the sense of intimacy and closeness [42,43].

The distinguishing feature of a high-quality intimate relationship is the couples’ ability to understand and support themselves. When we have needs, if our spouse can provide timely feedback, this can serve to indicate good intimacy [44]. Individuals should take positive measures to enhance interpersonal relationships and strengthen communication with each other. Positive intimacy can more effectively reduce risk behaviors and improve health behaviors [32,45,46].

### 4.3. Importance of the Type of Positive Intimacy in Couples’ Relationships

The above discussion fully illustrates the value and significance of the type of positive intimacy, which is considered a key factor in many categories of couples’ relationships [47,48,49].

According to the conservation of resources (COR) theory [50] and job demands–resources (JD-R) model [51], when individuals face a lack of resources, they reduce the cost of resources and preserve resources. Individuals with sufficient resources have the ability to obtain more resources and resource increments, and have stronger defense capabilities in the face of resource loss. On the contrary, resource-deficient individuals have a lower ability to acquire and maintain resources and are more vulnerable to the pressure of resource loss.

To cope with the loss of resources, individuals will make use of their remaining resources, one of which is the couples’ relationship [52,53]. When faced with external pressure (the early stage of the COVID-19 pandemic), people’s resources are limited and reduced, and relationships become a valuable resource during home quarantine. If individuals turn to intimacy to create an atmosphere of positive communication, build a good relationship with their spouse, and jointly deal with current difficulties, rather than using resource-consuming ways, such as conflict and blame, it will be a good way to counter the resource shortage [54,55].

To summarize, the conclusions of this study support the perspective that external stress can promote the improvement of couples’ relationships. Simultaneously, it can be predicted that a positive type of intimacy will help couples overcome the COVID-19 pandemic. Future research should focus on the types of positive intimacy and the role they play in couples’ relationships [23]. Of course, our research node is shortly after home quarantine, so the conclusions are more applicable to the early stages of the COVID-19 pandemic.

### 4.4. Limitations

There were several limitations to this study. First, we used an unvalidated self-report questionnaire. In subsequent quantitative research, the measurement tools can be standardized. Second, this study did not assess the stress level, and such variables as years of relationship with a partner or number of children have not been controlled, although some studies had shown that their effects are not obvious [21,24]. Third, the specific cultural context did not allow for the generalization of results to other populations. Follow-up studies should focus on people from different cultures to compare their differences. Finally, this study was limited by the current COVID-19 pandemic and thus used a cross-sectional research design. Thus, causality cannot be inferred. Future research should use longitudinal or experimental designs to explore the causal relationships.

## Data Availability

The data presented in this study are available on request from the corresponding author.
